# Soy isoflavones improve the oxidative stress induced hypothalamic inflammation and apoptosis in high fat diet-induced obese male mice through PGC1-alpha pathway

**DOI:** 10.18632/aging.103197

**Published:** 2020-05-13

**Authors:** Dejiang Pang, Chengcheng Yang, Qihui Luo, Chao, Li, Wentao Liu, Lixia Li, Yuanfeng Zou, Bin Feng, Zhengli Chen, Chao Huang

**Affiliations:** 1 Laboratory of Experimental Animal Disease Model, College of Veterinary Medicine, Sichuan Agricultural University, Chengdu 611130, P.R. China; 2 Neuroscience and Metabolism Research, State Key Laboratory of Biotherapy, West China Hospital, Sichuan University and Collaborative Innovation Center, Chengdu 610041, P.R. China; 3 Key Laboratory of Animal Disease and Human Health of Sichuan Province, College of Veterinary Medicine, Sichuan Agricultural University, Chengdu 611130, P.R. China; 4 Animal Nutrition Institute, Sichuan Agricultural University, Chengdu 611130, P.R. China

**Keywords:** soy isoflavones, obesity, oxidative stress, inflammation, PGC1-alpha

## Abstract

Obesity is a common metabolic disorder that increases the risk of many diseases, such as type II diabetes, hypertension, cardiovascular disease. Hypothalamus plays a very important role in the progression of obesity, and many studies reveal that hypothalamic injures are implicated in obesity processes. Here, we describe that the consumption of soy isoflavones, with a structural similarity to that of estradiol, could mitigate obesity through improving the hypothalamic inflammation and apoptosis, which are induced by oxidative stress. Also, our *in vitro* studies demonstrate that daidzein and genistein, common ingredients of soy isoflavones, could protect hypothalamic N42 cells against palmitic acid induced oxidative stress and apoptosis. Moreover, the transcriptional coactivator peroxisome proliferator-activated receptor γ coactivator 1 alpha (PGC1-alpha), which plays a role in oxidative defense, is increased after soy isoflavone treatment *in vivo* and *in vitro*, suggesting an improved effect of soy isoflavones on hypothalamic antioxidant defense is mediated by PGC-1α. Our study reveals a potential mechanism of soy isoflavones regulating oxidative stress induced hypothalamic inflammation and cellular apoptosis, which will be important for obesity treatment.

## INTRODUCTION

Obesity, as a serious public health problem, will greatly increase the risk of many diseases, such as type II diabetes, hypertension, cardiovascular disease, sleep apnea, psychological diseases and tumors [1–4]. The occurrence of obesity is caused by the imbalance in the homeostasis of the maintenance of body fat volume, which is caused by many reasons, including genetic susceptibility and the influence of environmental factors [5]. Among the environmental factors, the popularity and over-uptake of high-calorie diet play a very important role. Under the influence of this diet habit, the occurrence of obesity is not only with a high speed, but also with a lower age [6]. Therefore, it has very important scientific and clinical significance to carry out detailed and in-depth research on the mechanism of dietary induced obesity and to propose reasonable intervention and treatment methods on this basis.

Substantial literatures have reported that the body fat content is regulated by the homeostasis of the internal energy environment [7]. The brain plays a critical important role in this process, involving in areas from the cortex to the brainstem, and especially the hypothalamus is thought to be the key area [8]. In 2005, De Souza et al. first reported that there was occurrence of neuroinflammation in the hypothalamus region of obese rats induced by high-fat diet [9]. Moreover, the study of Thaler et al. shown that the onset of hypothalamic neuroinflammation was earlier than that of obesity, suggesting that hypothalamic neuroinflammation may be the key cause of obesity [10].

The same as neuro-inflammation, hypothalamic cellular apoptosis is also thought to be involved in high-fat diet induced obesity. Moraes et al. reported that consumption of high-fat diet could induce apoptosis of neurons in the arcuate nucleus and lateral hypothalamus [11]. However, oxidative stress is closely related to neuro-inflammation and cellular apoptosis [12]. The occurrence of oxidative stress in the brain has been reported in the obesity model. Morrison and Nerurkar et al. have detected large amounts of oxidative damage to proteins in the brains of obese mice induced by a high-fat diet [13, 14], and the antioxidant effects of estrogen made female mice significantly resistant to high-fat diet-induced neuroinflammation [15]. These studies suggest that hypothalamic oxidative stress may be implicated in the hypothalamic inflammation and cellar apoptosis induced by high-fat diet and subsequent occurrence of obesity. Therefore, it is of interests to explore the treatment of obesity from the aspect of hypothalamic oxidative stress.

Soy has been a part of the diet of many countries for centuries and is generally considered beneficial [16]. Soy isoflavones are the natural phytoestrogens in soy, which are considered to be the source of beneficial effects of soy, and have the potential to prevent many diseases, such as breast cancer, osteoporosis, hypercholesterolemia and especially obesity [17]. For example, the study of Luo et al. show that soy isoflavones decrease food intake and body weight gain in mice [18]. Moreover, soy isoflavones can regulate lipid metabolism through AKT/mTORC1 pathway [19] and improve the colonic immune function in obese rats induced by high-fat diet [20]. However, the effects of soy isoflavones on hypothalamus under obesity condition are not well defined, especially describing this from oxidative stress aspect. Here, we report that soy isoflavones improve the oxidative stress induced neuro-inflammation and cellular apoptosis of hypothalamus in diet-induced obesity (DIO) male mice through transcriptional coactivator peroxisome proliferator-activated receptor γ coactivator 1 alpha (PGC1-α), which plays a pivotal role in oxidative defense. Our research provides a new insight for soy isoflavones on obesity treatment, which will be important for its further application.

## RESULTS

### Soy isoflavones exhibit remarkable effects on body weight and adiposity in DIO male mice

**Figure 1:**
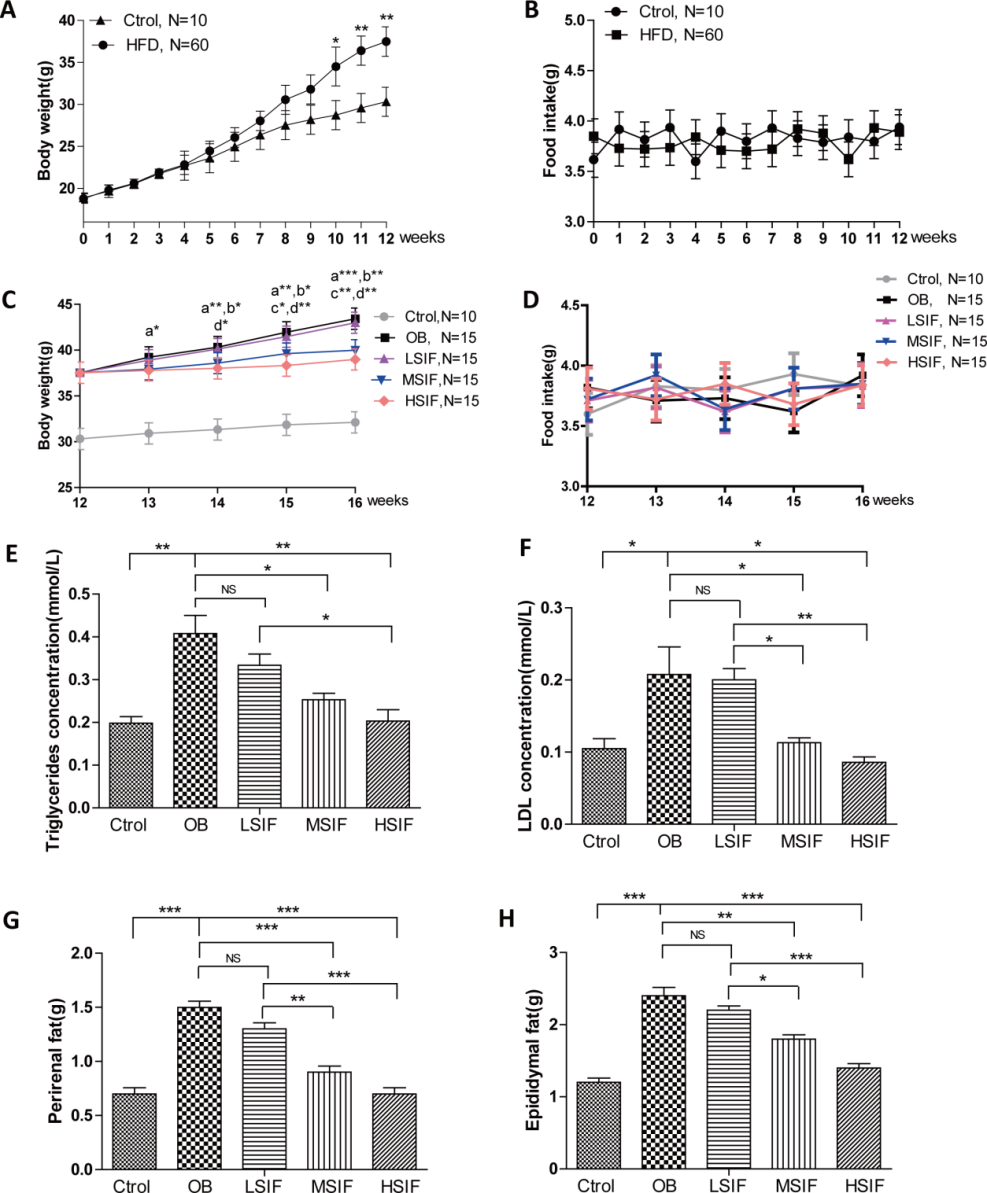
**Soy isoflavones reduce the body weight, the plasma TG and LDL concentrations as well as visceral fat weights in DIO male mice.** (**A**) Quantification shows the body weight of mice fed with basal diets and high fat diets; (**B**) Quantification shows no difference in food intake of mice fed with basal diets and high fat diets; (**C**) Quantification shows the body weight trend of DIO mice fed with basal diets and the addition with different doses of soy isoflavones. a: HSIF *vs.* OB; b:MSIF *vs.* OB. c: LSIF *vs.* MSIF; d: LSIF *vs.* HSIF. (**D**) Quantification shows the food intake trend of DIO mice fed with basal diets and the addition with different doses of soy isoflavones. (**E**) Quantification shows the triglycerides concentration of DIO mice feed with basal diets and the addition with different doses of soy isoflavones; (**F**) Quantification shows the LDL concentration of DIO mice fed with basal diets and the addition with different doses of soy isoflavones. (**G**) Quantification shows the perirenal fat weight of DIO mice fed with basal diets and the addition with different doses of soy isoflavones; (**H**) Quantification shows the epididymal fat weight of DIO mice fed with basal diets and the addition with different doses of soy isoflavones. ns, no statistical significance, * p < 0.05, ** p < 0.01, ***p < 0.001.

To investigate the role of soy isoflavones (SIF) in obesity, we generated the DIO male mice with high fat diets. 5 weeks old male C57BL/6J mice were fed with a high-fat diet or a normal diet. After twelve-week feeding, mice with high fat diets gained more body weight than the control ones (Figure 1A), and there was no statistical difference of food intake between the control mice and the DIO mice (Figure 1B). After the consumption of SIF, we found that the high dose of soy isoflavones (HSIF) and the middle dose of soy isoflavones (MSIF) could significantly reduce the body weight of DIO male mice, but there was no statistical difference of body weight between the low dose of soy isoflavones group (LSIF) and the obesity control group (OB group) (Figure 1C). Besides, food intakes were also no statistical difference among these groups (Figure 1D). Obese individuals have high levels of triglycerides (TG) and low-density lipoproteins (LDL) in their plasma, so we measured the levels of TG and LDL in plasma of these mice. We found a significant reduction of TG and LDL concentrations in the soy isoflavones groups compared with those in the OB group, especially in MSIF and HSIF ones (Figure 1E, 1F). Moreover, we found a significant reduction of perirenal fat and epididymal fat weight in the soy isoflavones groups compared with the OB group, also especially in MSIF and HSIF ones (Figure 1G, 1H). These data suggested that soy isoflavones could mitigate obesity in DIO male mice.

### Soy isoflavones could reduce neuroinflammation in hypothalamus of DIO male mice

**Figure 2:**
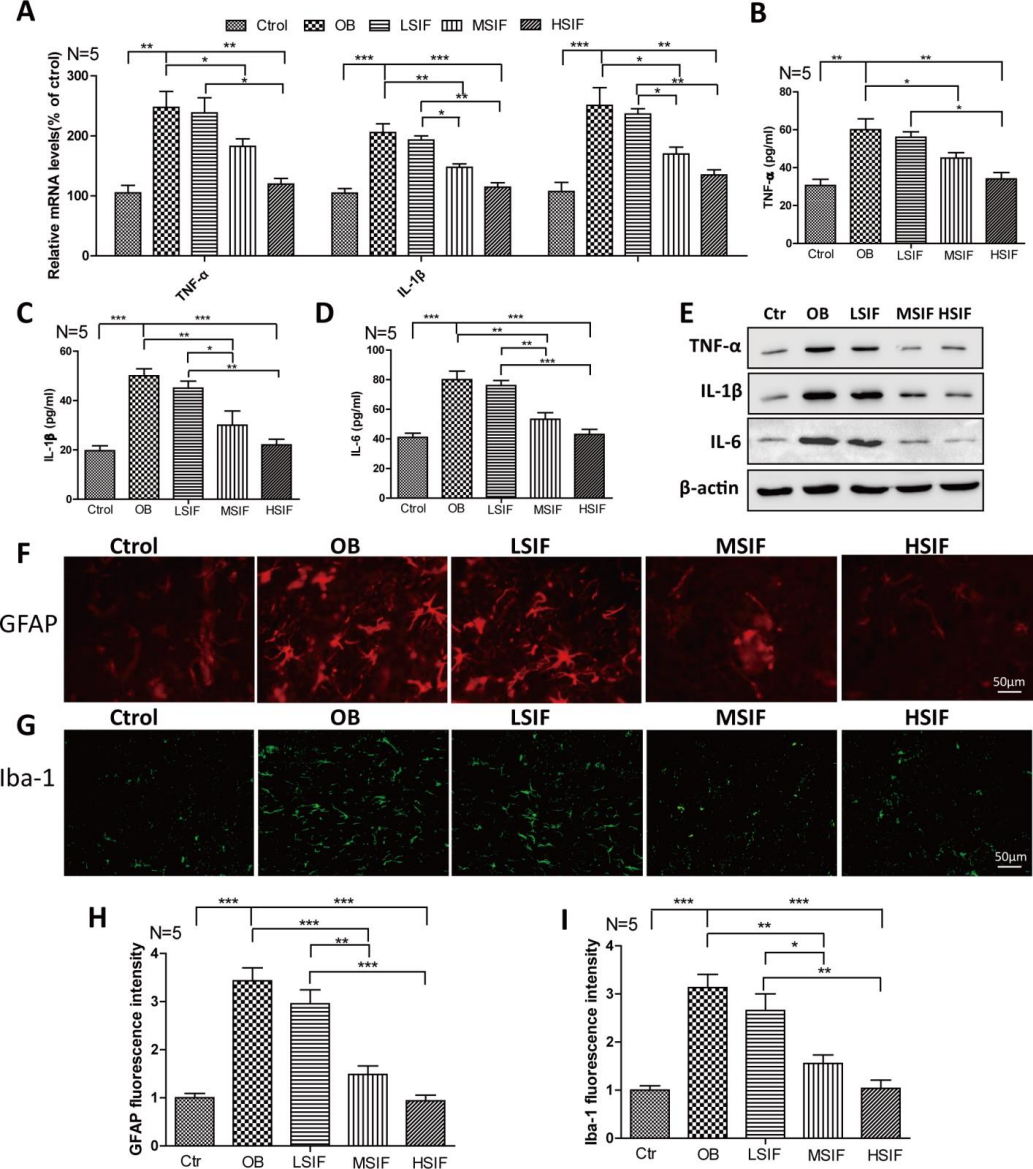
**Soy isoflavones reduce neuroinflammation in hypothalamus of DIO male mice.** (**A**) Quantification shows the mRNA levels of inflammatory cytokines in hypothalamus of DIO mice fed with basal diets and the addition with different doses of soy isoflavones; (**B**–**E**) Quantification shows the protein levels of inflammatory cytokines in hypothalamus of DIO mice fed with basal diets and the addition with different doses of soy isoflavones by ELISA assay; (**F**, **G**) Immunofluorescence staining shows the positive cells of GFAP were reduced in hypothalamus of DIO mice fed with different doses of soy isoflavones compared with the ones fed with basal diets. Scale bar: 50μm. (**H**, **I**) Immunofluorescence staining shows the positive cells of Iba-1 were reduced in hypothalamus of DIO mice fed with different doses of soy isoflavones compared with the ones fed with basal diets. * p < 0.05, ** p < 0.01, ***p < 0.001.

There was occurrence of neuroinflammation in the hypothalamus region of obese rats induced by high-fat diet, so we detected the inflammatory reactions in DIO male mice. We found that both the mRNA and protein levels of pro-inflammatory cytokines(TNF-α, IL-1β, IL-6) were increased in DIO male mice hypothalamus compared with control male mice, HSIF and MSIF could significantly reduce the levels of these factors compared with those in DIO male mice, but there was no statistical difference of the levels of inflammatory cytokines between the LSIF group and the OB group (Figure 2A–2E). Hypothalamic inflammation occurs with rapid activation of a complex network of cells, especially astrocyte and microglia [10]. As indicated in Figure 2F–2I, the numbers of activated astrocyte (GFAP positive cells) and microglia (Iba-1 positive cells) were significantly increased in DIO male mice hypothalamus compared with control male mice. HSIF and MSIF could significantly reduce the numbers of activated astrocyte and microglia of DIO male mice, but there was no statistical difference of the numbers of activated astrocyte or microglia between the LSIF group and the OB group. These results revealed that soy isoflavones could reduce neuroinflammation in hypothalamus of DIO male mice

### Soy isoflavones could reduce oxidative stress in hypothalamus of DIO male mice

**Figure 3:**
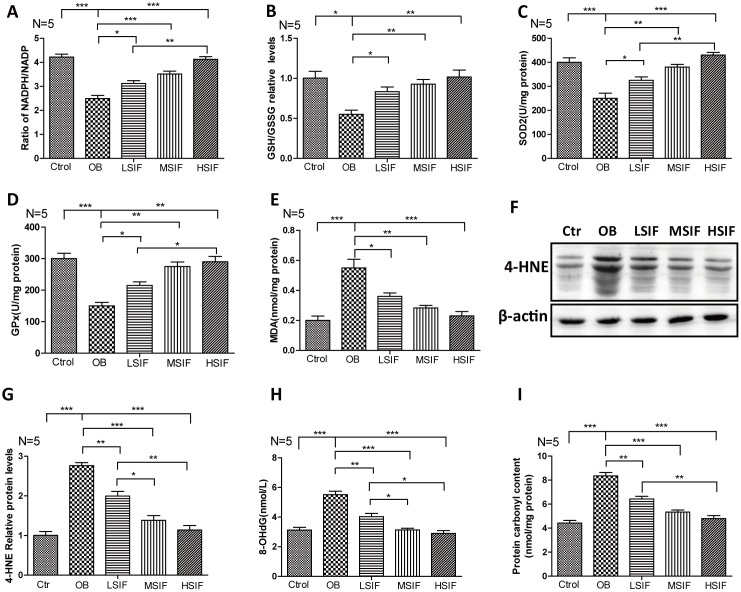
**Soy isoflavones reduce oxidative stress in hypothalamus of DIO male mice.** (**A**) Quantification shows the NADPH/NADP ratio in hypothalamus of DIO mice fed with basal diets and the addition with different doses of soy isoflavones; (**B**) Quantification shows the GSH/GSSG ratio in hypothalamus of DIO mice fed with basal diets and the addition with different doses of soy isoflavones; (**C**) Quantification shows the SOD2 activity in hypothalamus of DIO mice fed with basal diets and the addition with different doses of soy isoflavones; (**D**) Quantification shows the GPx activity in hypothalamus of DIO mice fed with basal diets and the addition with different doses of soy isoflavones; (**E**) Quantification shows the MDA levels in hypothalamus of DIO mice fed with basal diets and the addition with different doses of soy isoflavones; (**F**, **G**) Western blots and quantification show the 4-HNE levels in hypothalamus of DIO mice fed with basal diets and the addition with different doses of soy isoflavones; (**H**) Quantification shows the 8-OHdG levels in hypothalamus of DIO mice fed with basal diets and the addition with different doses of soy isoflavones; (**I**) Quantification shows the protein carbonyl contents levels in hypothalamus of DIO mice fed with basal diets and the addition with different doses of soy isoflavones. * p < 0.05, ** p < 0.01, ***p < 0.001.

Oxidative stress has been tightly associated with brain inflammation in overnutrition related diseases [21], so we analyzed the occurrence of oxidative stress in DIO male mice. We first measured the NADPH/NADP ratio to monitor the redox balance [22]. We found that the NADPH/NADP ratio was decreased in the hypothalamus of DIO male mice and significantly increased in the hypothalamus of DIO male mice after feeding with soy isoflavones (Figure 3A). Likewise, We found that the GSH/GSSG ratio was decreased in the hypothalamus of DIO male mice and also significantly increased in the hypothalamus of DIO male mice after feeding with soy isoflavones (Figure 3B). The activity of multiple antioxidant enzymes, such as superoxide dismutase 2(SOD2) and glutathione peroxidase (GPx), were decreased in the hypothalamus of DIO male mice and significantly increased in the hypothalamus of DIO male mice feeding with soy isoflavones (Figure 3C, 3D). The present of oxidative stress will result in accumulating oxidative damages. We found that the oxidative damage products of lipid—malondialdehyde (MDA) and 4-hydroxy-2-nonenal(4-HNE)- were increased in the hypothalamus of DIO male mice and significantly reduced in the hypothalamus of DIO male mice supplemented with soy isoflavones (Figure 3E–3G). Besides, 8-hydroxy-2 deoxyguanosine (8-OHdG) and protein carbonyl levels that indicate free radical oxidative damage to DNA and proteins were also significantly reduced in the hypothalamus of DIO male mice supplemented with soy isoflavones compared with DIO mal mice (Figure 3H, 3I). These results revealed that HFD could induce oxidative stress in the hypothalamus of male mice, and soy isoflavones could mitigate the redox unbalance and reduce the related oxidative injures.

### Soy isoflavones could reduce apoptosis in hypothalamus of DIO male mice

**Figure 4:**
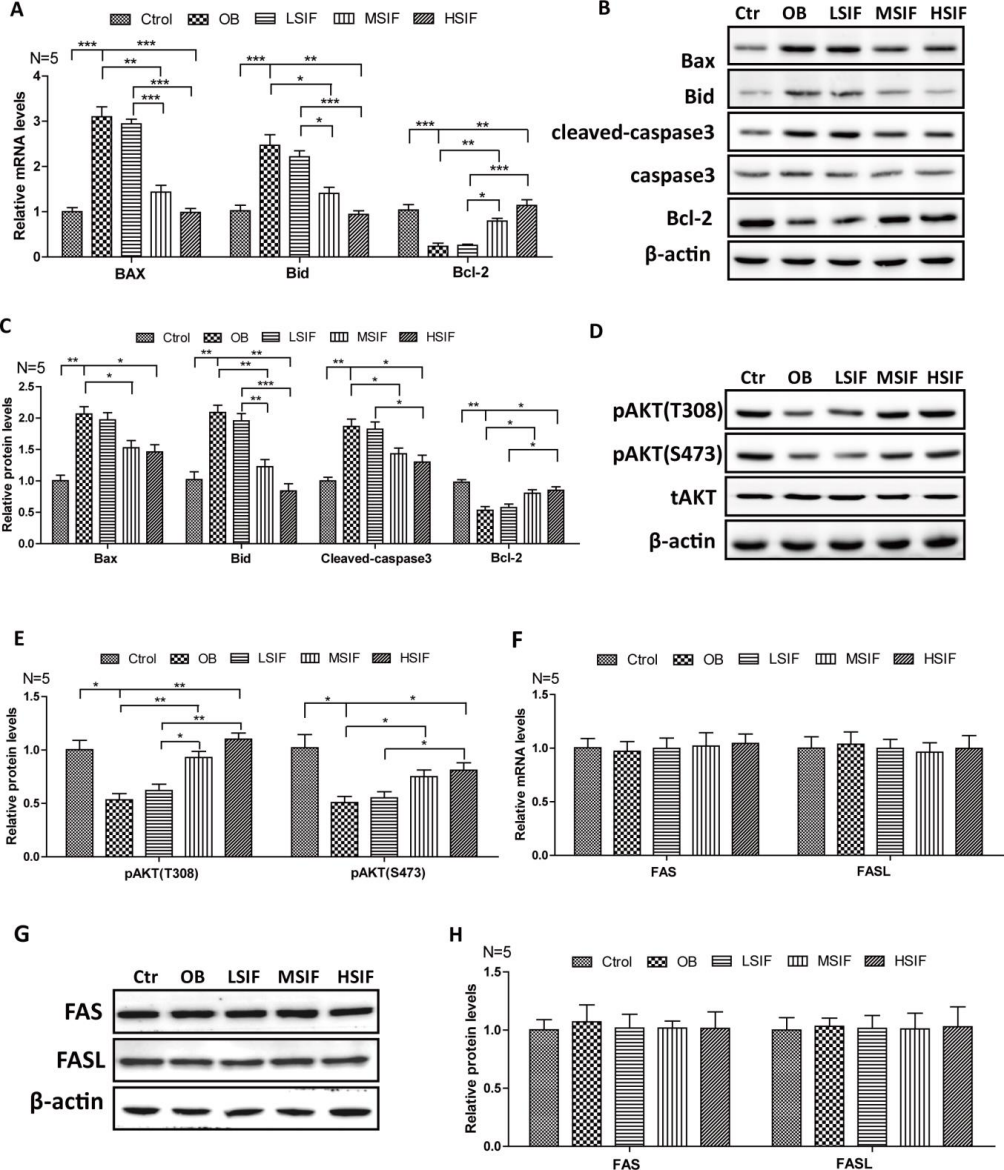
**Soy isoflavones could reduce apoptosis in hypothalamus of DIO male mice.** (**A**) Quantification shows the mRNA levels of apoptosis factors in hypothalamus of DIO mice fed with basal diets and the addition with different doses of soy isoflavones; (**B**, **C**) Western blots and quantification show the apoptosis factors protein levels in hypothalamus of DIO mice fed with basal diets and the addition with different doses of soy isoflavones; (**D**, **E**) Western blots and quantification show the phosphorylation levels of AKT in hypothalamus of DIO mice fed with basal diets and the addition with different doses of soy isoflavones; (**F**) Quantification shows the mRNA levels of FAS and FASL in hypothalamus of DIO mice fed with basal diets and the addition with different doses of soy isoflavones; (**G**, **H**) Western blots and quantification show the protein levels of FAS and FASL in hypothalamus of DIO mice fed with basal diets and the addition with different doses of soy isoflavones; * p < 0.05, ** p < 0.01, ***p < 0.001.

Many studies have shown that oxidative stress can induce apoptosis, so we detected the expressions of key factors regulating apoptosis. We found that the levels of pro-apoptotic factor mRNA (BAX and BID) were significantly increased in the hypothalamus of DIO male mice and significantly decreased in the hypothalamus of DIO male mice after feeding with soy isoflavones (Figure 4A). Conversely, we found that the levels of anti-apoptotic factor Bcl-2 mRNA was significantly decreased in the hypothalamus of DIO male mice and significantly increased in the hypothalamus of DIO male mice after feeding with soy isoflavones (Figure 4A). Consistent with this, accumulating protein levels of BAX, BID and cleaved caspase-3 were present in hypothalamus of OB mice, but were decreased after feeding with soy isoflavones. Meanwhile, increased protein levels of Bcl-2 were observed in SIF treated ones (Figure 4B, 4C). Furthermore, we found that the phosphorylation of AKT(Thr308 and Ser473 sites), which is the upstream regulator of Bcl-2/Bax pathway, was significantly decreased in the hypothalamus of DIO male mice and significantly increased in the hypothalamus of DIO male mice after feeding with SIF (Figure 4D, 4E). The death receptor signaling pathway is also a key regulator of apoptosis, and we found no changes of the mRNA levels, as well as the protein levels, of FAS and FASL both in the hypothalamus of DIO male mice and the SIF treated ones (Figure 4F–4H). These data suggested that HFD induced oxidative stress may trigger cellular apoptosis through the mitochondrial apoptotic pathway, and SIF could mitigate this injures in hypothalamus of DIO male mice.

### Soy isoflavones could improve palmitic acid-induced apoptosis in hypothalamic cell line

**Figure 5:**
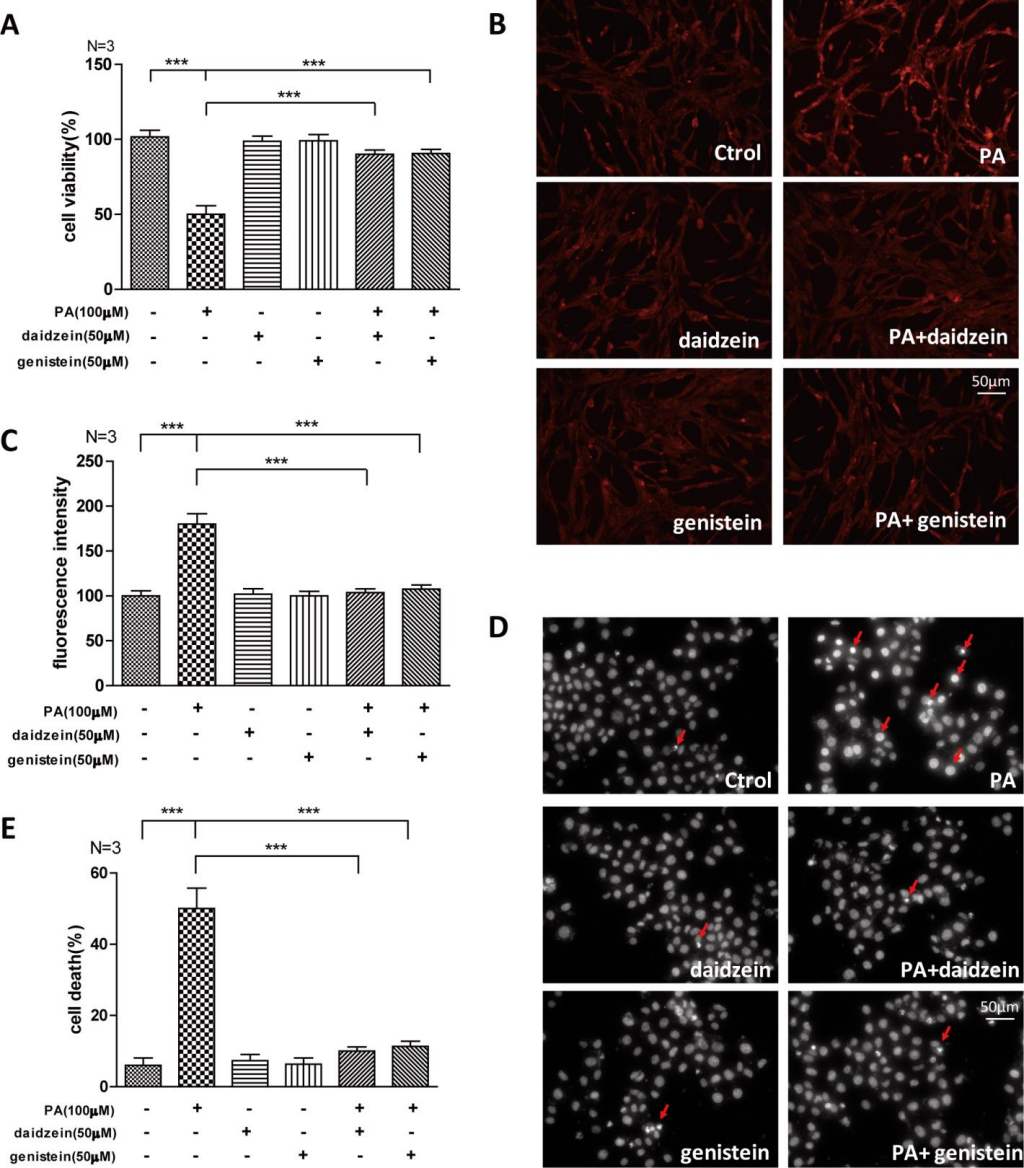
**Soy isoflavones could improve palmitic acid-induced apoptosis in hypothalamic cell line.** (**A**) Quantification shows the cell viability of N42 cells treated with PA(100μM), respectively, alone or in combination with daidzein(50μM) or genistein(50μM) for 24h. (**B**, **C**) Representative images and quantification show the ROS production of N42 cells under treatment with PA(100μM), respectively, alone or in combination with daidzein(50μM) or genistein(50μM) for 24h by dihydroethidium labeling. Scale bar: 50μm. (**D**, **E**) Representative images and quantifications show the nuclear morphology of N42 cells under treatment with PA(100μM), respectively, alone or in combination with daidzein(50μM) or genistein(50μM) for 24h. * p < 0.05, ** p < 0.01, ***p < 0.001. Scale bar: 50μm.

The saturated long-chain fatty acid palmitic acid (PA) is a major circulating saturated fatty acid, and the plasma concentration of PA is significantly increased in obesity subjects [23, 24]. PA was often used as a high fat inducer in vitro, we analyzed whether common ingredients of SIF -daidzein and genistein- could improve the oxidative stress induced by PA. We found that cell viability of N42 cells, which is an embryonic mouse hypothalamic cell line [25], was suppressed by PA, while significantly increased cell viability was detected by CCK-8 assay in N42 cells exposed to PA/daidzein or PA/genistein combined treatment (Figure 5A). Moreover, we found significantly increased reactive oxygen species (ROS) accumulation in N42 cells after PA treatment by fluorescent dihydroethidium (Eth) labeling, while ROS accumulation was decreased after daidzein or genistein treatment (Figure 5B, 5C). Excessive palmitic acid has been implicated in the induction of apoptosis in a large variety of cell types [26, 27]. We found that PA significantly induced apoptosis by Hoechst 33342 staining, while PA/daidzein or PA/genistein combined treatment can inhibit the apoptosis inducing by PA (Figure 5D, 5E). These results revealed that daidzein and genistein could protect hypothalamic cell line against oxidative stress-induced apoptosis under the treatment of PA, which was consistent with our *in vivo* study.

### Soy isoflavones could increase PGC1-alpha transcription in hypothalamus of DIO male mice

**Figure 6:**
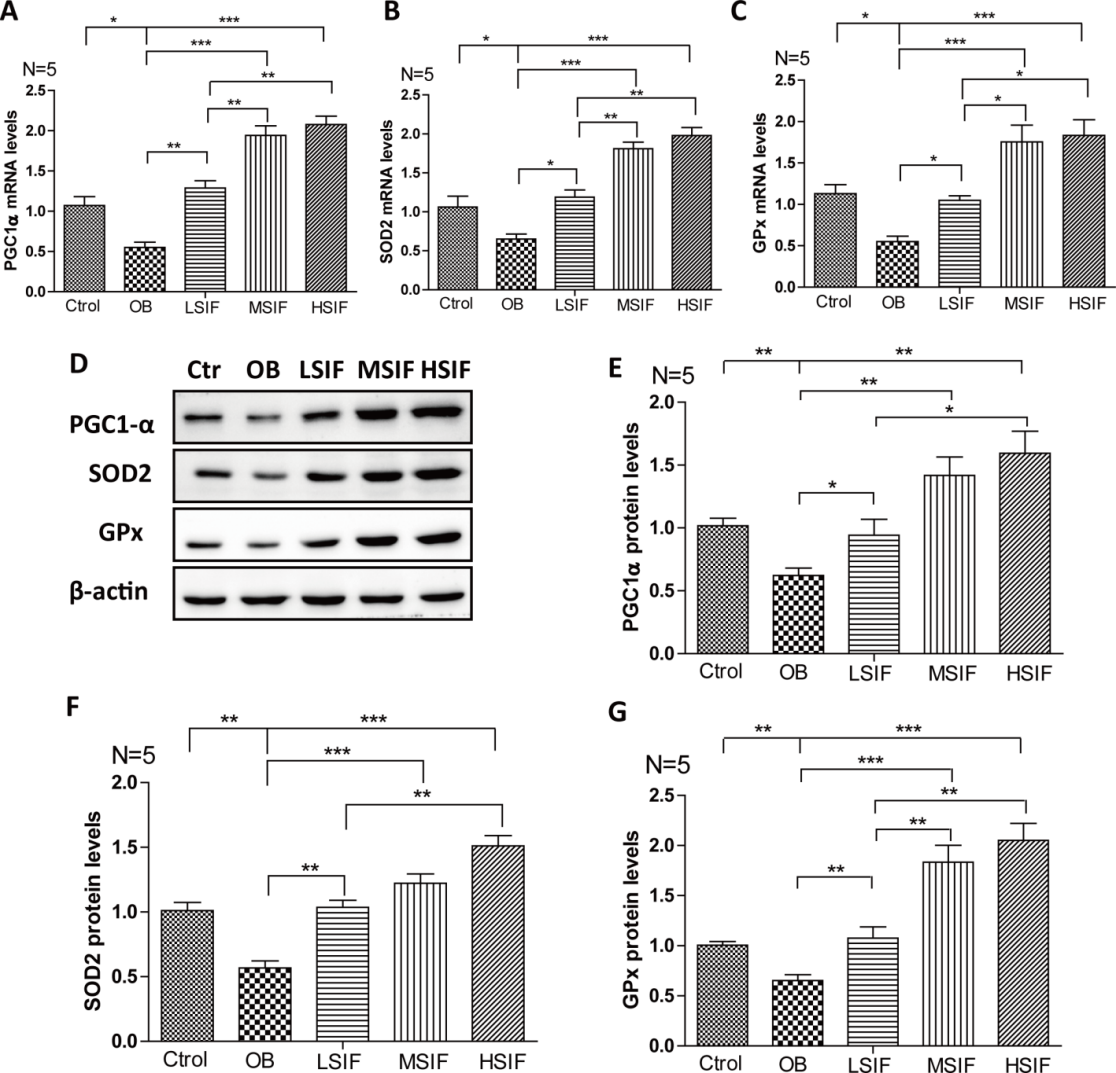
**Soy isoflavones increase PGC1-alpha transcription in hypothalamus of DIO male mice.** (**A**) Quantification shows the mRNA levels of PGC1-alpha in hypothalamus of DIO mice fed with basal diets and the addition with different doses of soy isoflavones; (**B**) Quantification shows the mRNA levels of SOD2 in hypothalamus of DIO mice fed with basal diets and the addition with different doses of soy isoflavones; (**C**) Quantification shows the mRNA levels of GPx in hypothalamus of DIO mice fed with basal diets and the addition with different doses of soy isoflavones; (**D**–**G**) Western blots and quantification show the protein levels of PGC1-alpha, SOD2 and GPx in hypothalamus of DIO mice fed with basal diets and the addition with different doses of soy isoflavones. * p < 0.05, ** p < 0.01, ***p < 0.001.

Through the above research, we found that soy isoflavones function to attenuate hypothalamic oxidative stress, but the mechanism behind is not clear. The transcriptional coactivator peroxisome proliferator-activated receptor γ coactivator 1 alpha (PGC1-α) plays a role in oxidative defense, and PGC1-α is a master regulator of ROS scavenging enzymes including manganese superoxide dismutase (SOD), glutathione peroxidase (GPx) and so on [28]. We found that the mRNA and proteins levels of PGC1-α were down-regulated in the hypothalamus of DIO male mice, and were up-regulated after feeding with soy isoflavones (Figure 6A, 6D, 6E). The downstream target gene of PGC1-α, SOD 2 (Figure 4B, 4D, 4F) and GPx (Figure 6C, 6D, 6G) maintained the same trend changed in the hypothalamus of DIO male mice and the mice feeding with soy isoflavones. These results suggested that soy isoflavones may induce PGC1-α transcription in hypothalamus of DIO male mice.

### Soy isoflavones could increase PGC1-alpha activity in hypothalamic cell line

**Figure 7:**
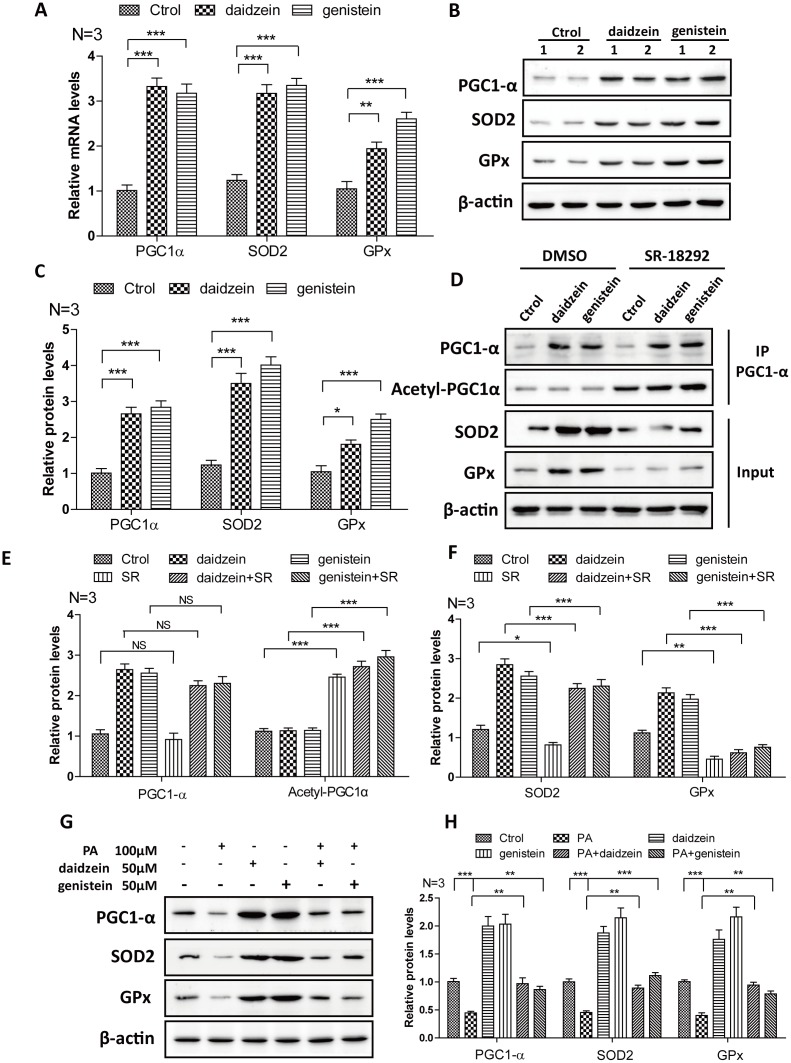
**Soy isoflavones increase PGC1-alpha activity in hypothalamic cell line.** (**A**) Quantification shows the mRNA levels of PGC1-alpha, SOD2 and GPx were increased under daidzein(50μM) and genistein(50μM) treatment for 24h in N42 cells; (**B**, **C**) Western blots and quantification show the protein levels of PGC1-alpha, SOD2 and GPx were increased under daidzein(50μM) and genistein(50μM) treatment for 24h in N42 cells; (**D**–**F**) Western blots and quantification show the SR-18292(20μM) administration inhibited the protein levels of SOD2 and GPx induced by daidzein and genistein treatment in N42 cells. (**G**, **H**) Western blots and quantification show the PA(100μM) administration inhibited the protein levels of PGC1-alpha, SOD2 and GPx, while daidzein and genistein could abolish the inhibition of PA in N42 cells. ns, no statistical significance, * p < 0.05, ** p < 0.01, ***p < 0.001.

In order to further convince the findings from *in vivo*, we evaluate the effects of daidzein and genistein on PGC1-α. We found that the mRNA (Figure 7A) and protein levels (Figure 7B, 7C) of PGC1-α were significantly increased after the addition of daidzein and genistein to N42 cells. The downstream target gene of PGC1-α, SOD2 and GPx were increased after the addition of daidzein and genistein to N42 cells (Figure 7A–7C). SR-18292 is a PGC-1α inhibitor, which increases PGC-1α acetylation, suppresses gluconeogenic gene expression and reduces glucose production in hepatocytes [29]. We found that SR-18292 can inhibit the increase of SOD2 and GPx inducing by daidzein and genistein (Figure 7D–7F). Furthermore, to evaluate whether the HFD influences PGC-1a in vitro, we analyzed PGC-1a expression following palmitic acid exposure. We treated N42 cells with PA and analyzed the PGC-1α expression. We found that the protein levels of PGC1-α, SOD2 and GPx were significantly reduced after the addition of PA, while daidzein and genistein rescued the reduction of PGC1-α, SOD2 and GPx inducing by PA (Figure 7G, 7H). These results revealed that daidzein and genistein could induce PGC1-α activity in hypothalamic cell line.

## DISCUSSION

Obesity is a big problem that medical, nutrition and public health professionals want to solve regardless of age, gender and occupation. The world health organization (WHO) has identified obesity as a disease and declared to the world that “obesity will be the number one global health problem” [30]. Over-uptake of high calorie and fat foods is thought to be the major reason for increasing incidence of obesity [31]. The beginning of the 20^th^ century, in the discovery of adiposogenital syndrome, the researchers gradually realized the close relationship between obesity and the hypothalamus injury. Babinski and Frohlich, et al. found that patients with hypothalamic tumors show signs of obesity and gonadal atrophy, suggesting that hypothalamic injury may lead to obesity [32, 33]. Smith, et al. removed the hypothalamus and pituitary gland of rats respectively, and found that only the absence of hypothalamus can lead to obesity of rat [34]. Moreover, many subsequent works have further clarified that hypothalamus injury will affect food intake and energy consumption, thus leading to body obesity [35–38]. Recently, hypothalamic inflammation and cellular apoptosis have been linked to the development and progression of obesity and its sequelae. Thaler et al. shown that the onset of hypothalamic neuroinflammation was earlier than that of obesity, suggesting that neuroinflammation may be the key cause of obesity [10]. Zhang, Valdearcos and Kleinridders et al. blocked the inflammation-related IKKβ /NF-κB signaling pathway in neurons, microglia cells and the whole central nervous system, respectively, and found that all of them could significantly inhibit obesity induced by high-fat diet [39–41]. In our study, we found that high-fat diet could induce severe hypothalamic neuroinflammation as indicated by increased expressions of inflammatory factors, as well as activated astrocyte and microglia. Then, the consumption of soy isoflavones can significant attenuate the neuroinflammation in the hypothallus of DIO male mice. Our finding is consistent with the previously reports that soy isoflavones participated in the anti-inflammatory process of brain. Ahmad, et al. reported that soy isoflavones could reduce the neuroinflammation in the brain of scopolamine-induced amnesia [42]. And, Ding et al. found attenuated inflammatory response and improved learning and memory ability in β-amyloid 1-42 treated rat supplemented with soy isoflavones [43]. But, our work first described the function of soy isoflavones in hypothalamic neuroinflammation response.

Although the relationship between hypothalamic neuroinflammation and the occurrence of obesity is widely reported, the mechanisms behind are not well defined. Oxidative stress is thought to be closely related to inflammation. From one aspect, reactive oxygen species (ROS) accumulating during oxidative stress are important signaling molecule that activates the inflammatory signaling pathway; From another aspect, inflammation response could generate more ROS that will aggravate oxidative stress [44–46]. During obesity, it is unknown whether hypothalamic oxidative stress is implicated in the progression of neuroinflammation, or the hypothalamic neuroinflammation is resulted from oxidative stress. In our study, we found accumulating oxidative damages in the hypothallus of DIO male mice, suggesting the induction of hypothalamic oxidative stress by high-fat diet. In addition, our study suggested that attenuated hypothalamic neuroinflammation by soy isoflavones supplementation should resulted from reduced oxidative stress. Because we found low dose of soy isoflavones could mitigate oxidative stress but not neuroinflammation in hypothalamus of DIO male mice. These data also suggested that high-fat diet induced hypothalamic neuroinflammation was dependent on the induction of oxidative stress.

In addition to neuro-inflammation, cellular apoptosis is also widely reported to be closely associated with oxidative stress in the brain [47]. In high-fat diet induced obesity models, widespread hypothalamic apoptosis was demonstrated [11]. The death receptor signaling pathway and mitochondrial apoptotic pathway are the major pathways that are implicated in the regulation of cellular apoptosis [48]. In our study, we found that high-fat diet could induce apoptosis through mitochondria related AKT/Bax/caspase-3 pathway, but not the death receptor signaling pathway. Besides, combined the data from *in vivo* and *in vitro*, our study suggested that oxidative stress may be responsible for the induction of hypothalamic apoptosis in DIO male mice, and we also demonstrated that SIF could mitigate this apoptotic process induced by high-fat diet. Moreover, we further demonstrated both in vivo and in vitro that the improvement function of soy isoflavones on hypothalamic antioxidant defense was mediated by the expression of PGC-1α, which is a master regulator of cellular antioxidant defense.

In conclusion, oxidative stress and neuroinflammation in the hypothalamus play an important role in the process of obesity, and our study found soy isoflavones can alleviate oxidative damage and neuroinflammation in the hypothalamus, thereby improving obesity symptoms in mice. Then, we demonstrated that high-fat diet could induce oxidative stress related hypothalamic apoptosis through mitochondrial apoptotic pathway, and SIF protect form this injure also through the regulation of oxidative stress. Finally, we reported that soy isoflavones could induce the expression PGC1-α in the hypothalamus that will further activate and up-regulate downstream antioxidant enzymes to protect cells against oxidative damage and further alleviate neuroinflammation and cellular apoptosis. Unfortunately, we can’t provide enough details to correlate the test concentrations of soy isoflavones with human exposure levels, which is the limitation of our experiments. But, our study provides new aspect on oxidative stress, neuroinflammation, cellular apoptosis and obesity, which is important for the treatment of obesity.

## MATERIALS AND METHODS

### Animal care and maintenance

All animal work was done in accordance with the Animal Care and Use Committee Guidelines of Sichuan Agricultural University, China. Eighty male C57BL/6J mice (4 weeks old) were purchased from Chengdu dashuo experimental animal co. LTD and were maintained in individual cages in a specific pathogen-free environment. After 1 week of acclimatization, they were randomly divided into two groups, one group were fed with the basal diet (Research Diets, D10001), another group were fed with the high fat diet (Research Diets, 60% fat (kcal), D12492). These mice were continuously treated with the indicated diets for 12 weeks to induce obesity and the body weight was measured weekly. Then, DIO male mice were randomly divided into four groups, obesity control group (OB) and three obesity soy isoflavones group. They were fed with the high-fat diet with different doses of soy isoflavone additions (soy isoflavone extracts, North China Pharmaceutical Co., Ltd., Shijiazhuang, China) as described in Table 1. The compounds of soy isoflavone extracts, as quantified by HPLC, are shown in Table 2. After four weeks of feeding, these mice were decapitated for the subsequent experiments.

**Table 1:** The treatments of soy isoflavones on DIO male mice.

**Groups**	Control group (Ctr, n=10)	Obesity group (OB, n=15)	Low-dose soy isoflavones (LSIF, n=15)	Middle-dose soy isoflavones (MSIF, n=15)	High-dose soy isoflavones (HSIF, n=15)
**Diets**	Basal diets	High-fat diets	High-fat diets + 15mg/kg soy isoflavones	High-fat diets + 30mg/kg soy isoflavones	High-fat diets + 60mg/kg soy isoflavones

**Table 2:** Primers used for the real-time PCR analysis.

**Gene**	**Primers (5' -> 3')**
TNF-α	Forward Primer CCCTCACACTCAGATCATCTTCT
	Reverse Primer GCTACGACGTGGGCTACAG
IL-1β	Forward Primer GCAACTGTTCCTGAACTCAACT
	Reverse Primer ATCTTTTGGGGTCCGTCAACT
IL-6	Forward Primer TAGTCCTTCCTACCCCAATTTCC
	Reverse Primer TTGGTCCTTAGCCACTCCTTC
BAX	Forward Primer TGAAGACAGGGGCCTTTTTG
	Reverse Primer AATTCGCCGGAGACACTCG
BID	Forward Primer GCCGAGCACATCACAGACC
	Reverse Primer TGGCAATGTTGTGGATGATTTCT
BCL-2	Forward Primer GTCGCTACCGTCGTGACTTC
	Reverse Primer CAGACATGCACCTACCCAGC
PGC1-α	Forward Primer TATGGAGTGACATAGAGTGTGCT
	Reverse Primer CCACTTCAATCCACCCAGAAAG
FAS	Forward Primer TATCAAGGAGGCCCATTTTGC
	Reverse Primer TGTTTCCACTTCTAAACCATGCT
FASL	Forward Primer TCCGTGAGTTCACCAACCAAA
	Reverse Primer GGGGGTTCCCTGTTAAATGGG
SOD2	Forward Primer CAGACCTGCCTTACGACTATGG
	Reverse Primer CTCGGTGGCGTTGAGATTGTT
GPx-1	Forward Primer AGTCCACCGTGTATGCCTTCT
	Reverse Primer GAGACGCGACATTCTCAATGA
β-actin	Forward Primer GGCTGTATTCCCCTCCATCG
	Reverse Primer CCAGTTGGTAACAATGCCATGT

### Body weight, food intake, and plasma measurements

We measured the weight and food intake of mice weekly. Blood samples were collected from the lateral caudal vein. We used a sterile scalpel blade to make a 1- 2 mm incision at the tip of the tail, then blood was squeezed from the bottom of the tail to the top until a sufficient volume of blood was collected, and plasma triglycerides (TG) and low-density lipoproteins (LDL) were performed for biochemical analysis(Beckman CX4, Indianapolis, IN, USA). All groups were measured at fourth week after additional feeding with SIF.

### ELISA assay

ELISA assay kit purchased from Beyotime Institute of Biotechnology was used according to the manufacturer’s instructions. The mice were sacrificed by decollation. The hypothalamus was homogenized in PBS (pH 7.4) and then centrifuged. The supernatant was recovered and used as the test sample. Monoclonal capture antibodies specific to mouse TNF-α, IL-6, and IL-1β have been precoated on the plate, and mouse TNF-α, IL-6, and IL-1β binds to the capture antibody when added to the standard or sample. Then after a series of steps according to the manufacturer’s instructions, the absorbance of each well was recorder at 450 nm.

### Immunohistochemistry assay

Mice were anesthetized with 4% chloral hydrate and transcardially perfused with ice-cold 4% paraformaldehyde (PFA). The brains were removed and post-fixed overnight in 4% PFA and dehydrated in 30% sucrose at 4°C. Brains were cutted into 10-20 micron flake by frozen section. Standard immunohistochemistry was carried out to examine activation of astrocyte using anti-GFAP antibody (Millipore, MAB360), activation of microglia using anti-Iba-1 antibody (Wako, 019-19741).

### NADPH/NADP ratio assay

The NADPH/NADP ratio assay was performed on hypothalamus extracts using the NADP/NADPH assay kit (BioVision). According to the instruction manual, 5mg samples were extracted in 100μl of the recommended extraction buffer, and 50μl were processed following instructions. The OD450 values were measured by a plate-reader (Thermo), and the data was converted to nmol/sample using a standard curve and values were used for ratio as previously reported.

### Ratio of GSH/GSSH assay

The ratio of GSH/GSSH was measured using the GSH and GSSG Assay Kit (S0053) which was purchased from Beyotime Institute of Biotechnology according to the manufacturer’s instructions. Briefly, the hypothalamus was frozen with liquid nitrogen and then grounded into powder. For every 10 mg of ground tissue powder, added 30μl M solution to remove protein and fully vortex. Then added 70μl M solution and used glass homogenizer to fully homogenize. The sample was placed at 4 °C for 10 minutes and centrifuged at 10,000g 4 °C for 10 minutes. The supernatant was taken for the determination of glutathione. After the GSH/GSSH ratio of each group was measured, each experimental group was compared with the control group to get a relative value and quantified by GraphPad Prism software version 5.0 (GraphPad Software, Inc., La Jolla, CA, USA).

### Oxidative damage detection

The hypothalamus was homogenized in 200 μl ice-cold 0.86% saline buffer (1:9, weight/ volume) using an JY92-IIN ultrasonic cell disruptor (Xinzhi biotechnology co., Ltd., China) and then centrifuged at 3,000 rpm for 10~15 min at 4°C to obtain supernatants. 8-OHdG (Shanghai Enzyme-linked Biotechnology Co., Ltd, Shanghai, China), MDA, SOD, GPx, protein carbonyl were determined with clinical chemistry assay kits (Nanjing Jiancheng Bioengineering Institute, Nanjing, China) according to the manufacturer’s instructions.

### Cell culture

The N42 cell line is an embryonic mouse hypothalamic cell line (mHypoEN42) obtained from Cellutions Biosystems Inc. (Toronto, ON, Canada) and were cultured in Dulbecco modified Eagle medium (DMEM; Gibco, Waltham, MA, USA) supplemented with 10% fetal bovine serum (FBS; Gibco) and 1% penicillin-streptomycin (Gibco). Daidzein or genistein was added to the cultures at the final concentration of 50 μM for 24 h, then cells were collected to perform western blot or quantitative real-time PCR. In order to study the induction effect of soy isoflavones on PGC1-α, N42 cells were treated with 50 μM daidzein or genistein, alone or in combination with SR-18292 (20μM) for 24 h. The brief description about the PGC1-α acetylation detection in N42 cells is as follows: N42 cells were collected in 1% Triton X-100 buffer (containing protease inhibitor cocktail and phosphatase inhibitor cocktail) after treatment with 50 μM daidzein or genistein, alone or in combination with SR-18292. PGC1-α was immunoprecipitated overnight at 4°C by PGC1-α antibody and then performed the western blot to detect the acetylation level of PGC1-α by using the antibody anti-acetylated-lysine.

### Hoechst staining

Briefly, N42 cells were plated in 12-well plates with a density of 5x10^4^ cells/well and incubated overnight in DMEM with 10% FBS at 37°C. Then cells were treated with PA(100 μM), respectively, alone or in combination with daidzein(50 μM) or genistein(50 μM) for 24h. Next, the cells were washed in PBS three times and incubated in Hoechst33342 staining solution (10μg/ml) for 30min at 4°C. Finally, fluorescence microscopy was preformed to observe the nuclear changes of N42 cells. For each treatment group, ≥1,000 cells were analyzed in triplicate.

### Reactive oxygen species (ROS) detection

ROS assay kit purchased from Beyotime Institute of Biotechnology was used according to the manufacturer’s instructions. N42 cells were treated with PA(100 μM), respectively, alone or in combination with daidzein(50 μM) or genistein(50 μM) for 24h. Then, cells were incubated with 10 μM dihydroethidium (Eth) in DMEM at 37°C for 30 minutes. Finally, fluorescence microscopy was used to observe the ROS production of N42 cells. The fluorescence intensity of Eth was analyzed by image-pro plus software, then the fluorescence intensity value of each experimental group was compared with the fluorescence intensity value of the control group to get a relative value and quantified by GraphPad Prism software version 5.0 (GraphPad Software, Inc., La Jolla, CA, USA).

### Real-Time PCR

Total RNA was extracted from hypothalamus and N42 cells using Trizol reagent (Invitrogen; Thermo Fisher Scientific, Inc.), according to the manufacturer’s instructions. In each group, 2μg RNA were reverse transcribed into cDNA using the Revert Aid First Strand cDNA Synthesis kit (Thermo Fisher Scientific, Inc.) for RT-PCR as described before. One microliter cDNA was added to a total volume of 20μl PCR amplification system qPCR was performed using the SYBR Premix Ex Taq™ II kit (Takara Biotechnology, Co., Ltd., Dalian, China). Relative fold levels were determined using β-actin gene as normalizer control. The qPCR results were analyzed by Bio-Rad CFX Manager 3.0 software, and the normalized value of each experimental group was compared with the normalized value of the control group to get a relative value. Threshold cycle (Ct) values should be within the range mean±1 for each reference gene across all samples to ensure similar cDNA yield from each RT reaction. Primer sequences for SYBR Green probes of the target genes are described in Table 3.

**Table 3:** Composition of the soy isoflavone extracts.

**Compounds**	Daidzin	Glycitin	Genistein	Daidzein	Genistin	Total isoflavones
**Content**	50.98%	30.36%	8.80%	1.24%	0.06%	91.64%

### Western blotting analysis

Western blotting was performed according to standard procedures as reported before. To extract protein from cultured cells were sonicated in lysis buffer (2% SDS with proteinase inhibitors and phosphatase inhibitor). The protein concentration of each extract was measured using the BCA Protein Assay kit (Thermo Scientific Pierce). Equal amounts of protein from each extract were loaded into each lane of a gel and separated by SDS PAGE. The proteins were transferred onto PVDF membranes using standard procedures. The membranes were then blocked with 5% non-fat dry milk in TBST (TBS with 0.1% Tween 20, pH 7.6) for 1 hour at room temperature (RT) and incubated overnight with respected primary antibody at 4 °C. After 3 washes with TBST at RT for 10 minutes each wash, the membranes were incubated 1 hour with a 1:10000 dilution of appropriate secondary antibodies diluted in TBST at RT. The membranes were washed another 3 times with TBST at RT for 10 minutes each wash, proteins were then detected with ECL reagent (Thermo Scientific Pierce) and the membranes were exposed to film (Kodak). The antibodies in our experiments include: Bax (Abcam, ab232479, 1:1000 dilution), BID (Abcam, ab62469, 1:1000 dilution), Cleaved Caspase-3 (Abcam, ab32042, 1:300 dilution), Bcl-2 (Abcam, ab32124, 1:1000 dilution), AKT(Cell Signaling Technology, 9272S, 1:2000 dilution), pAKT (S473) (Cell Signaling Technology, 4060S, 1:2000 dilution), pAKT (T308) (Cell Signaling Technology, 2965S, 1:2000 dilution), FAS(Abcam, ab133619, 1:1000 dilution), FASL(Abcam, ab15285, 1:1000 dilution),4-HNE (Abcam, ab46545, 1:500 dilution), PGC1-α (Santa Cruz Biotechnology, sc-13067,1:200 dilution), acetylated-lysine (Cell Signaling Technology,9441, 1:1000 dilution), SOD2 (Abcam, ab68155, 1:2000 dilution), GPx-1 (Abcam, ab108427, 1:500 dilution), β-Actin (Boster, BM0627, 1:1000 dilution), Goat anti-Rabbit IgG (H+L) Secondary Antibody (pierce, 31460, 1:10000 dilution), Goat anti-Mouse IgG (H+L) Secondary Antibody (pierce, 31430, 1:10000 dilution). We used Image J software to quantitate western blots. Firstly, we measured the grey value of blots by using Image J. Secondly, normalized each sample using a normalizer (housekeeping protein such as β-actin, as mentioned above), then the normalized value of each experimental group was compared with the normalized value of the control group to get a relative value. Finally, we used GraphPad software to make a histogram.

### Statistical analysis

Data represent the mean and standard error of the mean (SEM). One-way ANOVA and post hoc tests were performed for all statistical significance analysis using GraphPad Prism software (GraphPad Software, Inc., La Jolla, CA, USA). *p < 0.05, **p < 0.01, ***p < 0.001.
